# Prevalence and associated factors of disordered eating among university and college students: A systematic review of Malaysian studies

**DOI:** 10.51866/rv.1019

**Published:** 2026-07-02

**Authors:** Cheong Lieng Teng, Mohamed Abdelwahab Eltanahy, Paramannantha Veloo Tesini, Hui Chii Voon, Sherreen Elhariri

**Affiliations:** 1 Department of Family Medicine, IMU University, Seremban, Negeri Sembilan, Malaysia.; 2 Department of Paediatrics, IMU University, Seremban, Negeri Sembilan, Malaysia.; 3 Department of Psychiatry, IMU University, Seremban, Negeri Sembilan, Malaysia.; 4 House Officer, Ministry of Health, Malaysia.; 5 Department of Surgery, IMU University, Seremban, Negeri Sembilan, Malaysia.

**Keywords:** Disordered eating, Malaysia, Prevalence, Systematic review, Young adult

## Abstract

**Introduction:**

University education is a stressful period in young adults’ lives, and the emergence of disordered eating during this time adds to the psychological morbidity that university students often experience and may need early identification and appropriate management. This systematic review of relevant Malaysian studies aimed to determine the pooled prevalence of disordered eating among university and college students and assess the associated factors.

**Methods:**

A comprehensive search for Malaysian studies using the Eating Attitudes Test (EAT- 26) was conducted using several bibliographic databases (PubMed, Scopus and Google Scholar). Relevant literature was systematically selected; pertinent data were extracted; and data on disordered eating (EAT-26 score of ≥20) and its associated factors were synthesised.

**Results:**

Twenty-one Malaysian cross-sectional studies that measured disordered eating using the EAT-26 were included. The pooled prevalence of disordered eating was 16.45% (random effects model, 95% CI=13.86–19.22), with a significantly higher odds ratio in female students (1.45, 95% CI=1.28–1.65). Various body image measures were assessed in nine studies, five of which reported a positive association between disordered eating and body dissatisfaction.

**Conclusion:**

One in six Malaysian university or college students, especially female students, may be affected by disordered eating, and many of them may also have body image concerns and body dissatisfaction. Disordered eating is a rising public health concern in the student population of colleges and universities and deserves further exploration of its potential adverse health outcomes.

## Introduction

Eating disorders are serious psychiatric morbidities that are portrayed by distortion in eating behaviours and weight regulation, resulting in significant physical illness and psychosocial impairment.^1^ Both the Fifth Edition of the Diagnostic and Statistical Manual of Mental Disorders (DSM-5) and 10 th Revision of the International Classification of Diseases (ICD-10) include six feeding and eating disorders, most of which occur more frequently among adolescents and young adults. Two subtypes of eating disorders (anorexia nervosa and bulimia nervosa) have increased mortality risks, especially among individuals requiring inpatient care.^2^

In their meta-analysis of international data on disordered eating among university students, Alhaj et al. reported a pooled prevalence of 24%.^3^ This review was based on 105 studies using 15 different rating scales, with the most commonly used scale being the 26-item Eating Attitudes Test (EAT-26). The EAT-26 is a self-administered questionnaire developed by Garner et al., and its original version comprised 40 items.^4^ In a validation study of the Eating Disorder Examination Questionnaire, Mohd Taib et al. reported a good overall internal reliability (Cronbach a=0.81) of the EAT-26 Malay version.^5^

Within the Asian context, there is growing evidence that the prevalence of disordered eating is increasing, particularly in regions undergoing rapid socio-cultural transitions.^6^ Understanding the changing socio-cultural influences of this trend has important public health implications, given the increasing frequency of body dissatisfaction among young adults. University students are a particularly vulnerable group, as this developmental period is characterised by heightened academic stress, lifestyle changes and greater autonomy, which may contribute to the onset or exacerbation of disordered eating behaviours.

The extensive review by Alhaj et al. included only four Malaysian studies.^3^ A preliminary literature review of Malaysian studies identified many more local studies not included in the review by Alhaj et al. Some of the reviewed local studies reported a fairly high prevalence of disordered eating among university students, and several studies suggested the need for early identification or screening of this problem among this population.^7,8^ In their review, Peat and Feltner highlighted the important role of primary care in the early detection of eating disorders but cautioned regarding the potential benefits and harms of routine screening in this setting.^9^

In view of the above-indicated considerations, we conducted a systematic review of relevant Malaysian studies to determine the pooled prevalence of disordered eating among university students, identify the associated factors and explore the challenges of the early detection of this problem.

## Methods

### Protocol registration

The protocol of this systematic review was registered in the International Platform of Registered Systematic Review and Meta-Analysis Protocols.^10^

### Information sources and search strategy

We searched PubMed and Scopus using a combination of the following search terms: ‘eating disorder’, ‘disordered eating’, ‘EAT-26’, ‘Eating Attitude Test’, ‘students’, ‘university’ and ‘Malaysia’ on 3 April 2026. These searches were supplemented by a Google Scholar search. Appendix 1 details the search strategy. Journal articles published from the inception of the databases to 31 December 2025 were retrieved.

### Eligibility criteria

The following studies were considered eligible for inclusion:

- Cross-sectional studies conducted in Malaysia.
- Studies that measured disordered eating using the EAT-26.
- Studies that included university or college students as their participants.

We excluded the following publications: books, monographs, reports, case reports, conference abstracts, editorials, letters, comments, reviews (narrative or systematic), study protocols, theses and dissertations.

### Selection process

The references were processed using EndNote 20 citation manager (Philadelphia, PA: Clarivate Analytics; 2025). The screening was conducted by two investigators.

### Quality assessment

The quality of the included studies was assessed by a pair of investigators using a checklist for prevalence studies published by the Joanna Briggs Institute (JBI).^11^ Disagreements between investigators were resolved through discussion with a third investigator.

### Data extraction and data items

The main outcome measure was the prevalence of disordered eating (by extracting counts of participants qualifying for this condition, the total sample size and sex-based data). When the counts or percentages were not reported (involving three studies), we recalculated the number of participants scoring EAT>20 using the method of “Normal Distribution Estimate”. In brief, we assume the scores were normally distributed, then we computed the z score for each gender and finally estimated the number of participants scoring above the threshold using the standard normal table. We also retrieved other data such as counts of participants by sex and study setting (public or private university). We noted whether the threshold of the EAT-26 score was as recommended by Garfinkel and Newman (score of ≥20).^12^ Risk factors of disordered eating, when reported, were also extracted. All relevant data in the included studies were extracted by a pair of investigators.

### Synthesis methods

A meta-analysis of prevalence data on disordered eating was performed using the MedCalc Statistical Software version 23.2.6 (Ostend, Belgium: MedCalc Software Ltd; 2025). Fixed-effects model was selected when study heterogeneity (I2) was less than 50%; otherwise, random-effects model was used. We investigated the study heterogeneity through sensitivity analysis, meta-regression and subgroup analysis using JASP (version 0.96, University of Amsterdam, Netherlands; 2026) and MedCalc. The variables evaluated in these analyses were as follows: sensitivity analysis: threshold of the EAT-26 score; meta-regression: threshold of the EAT-26 score, sampling method and percentage of female participants; and subgroup analysis: study setting.

This systematic review was prepared following the PRISMA guidelines.^13^

## Results

### Study selection

**Figure 1:**
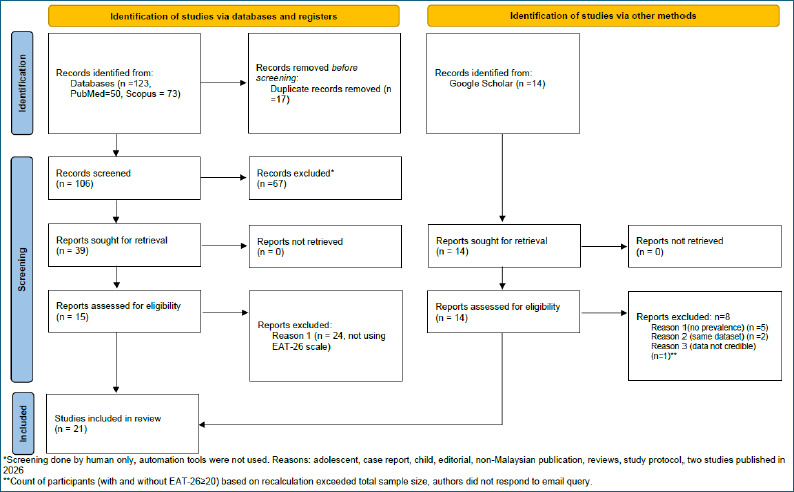
PRISMA flow diagram.

As shown in Figure 1, our comprehensive search identified 29 eligible studies. After excluding eight studies (see reasons within Figure 1), we included 21 studies in the qualitative and quantitative analyses.

### Study characteristics

The characteristics of the 21 included studies (total number of participants=9408) are presented in Table 1. Although most studies used the recommended threshold of an EAT-26 score of ≥20, some used a threshold of >20 (n=3) or did not mention the threshold (n=3). In 14 studies, students in various disciplines were recruited; three studies recruited only medical or health sciences students; and four studies did not specify the type of disciplines. All studies adopted a cross-sectional design; two of them were also validation studies of specific questionnaires. The quality score based on the JBI tool of these 21 studies varied from 4 to 9; some of the low scores were due to a lack of representativeness of the study sample (only three studies mentioned that random sampling was used), a lack of details on the study sample and the absence of the response rate (the response rate was reported in seven studies). As the prevalence data appeared to be measured appropriately, we included all of them in our analyses.

**Table 1:** Characteristics of the included studies.

Study	EAT-26 criteria	Setting and participant	Risk factor^*^ (name of the questionnaire)	JBI score
Abdalla et al. 2020^14^	≥20	One private university, students in various disciplines	High body mass index and high waist-to- height ratio, female	7
Azizi et al. 2020^15^	NA	Four public universities, students in various disciplines	EAT-26 score not associated with social media use but correlated with body image concern (Body Shape Questionnaire) (r=0.616)	6
Azli et al., 2024^16^	>20	One public university, students in various disciplines	Higher body image avoidance score in those with disordered eating (Body Image Avoidance Questionnaire) Sex, BMI and physical activity not associated	6
Azman et al., 2024^17^	≥20	One public university, health sciences students	Low self-esteem	6
Azman et al., 2022^7^	≥20	One private university, students in various disciplines	Monthly allowance of >RM 1000 Sex and ethnicity not associated	7
Chan et al., 2020^18^	≥20	One public university, students in various disciplines	Trying to lose weight, post-traumatic stress disorder (Breslau’s 7-item scale) Sex, ethnicity and depression not associated	8
Cheah et al., 2024^8^	≥20	20 universities/colleges, students in various disciplines	Female; perceived socio-cultural pressure, drive for muscularity, perfectionism Ethnicity, BMI and self-esteem not associated	8
Chin et al., 2020^19^	≥20	Seven universities in Kuala Lumpur and Selangor, students in various disciplines	EAT-26 score correlated with depression (CES-D), body dissatisfaction (Figure Rating Scale) and negative body appreciation (Body Appreciation Scale)	7
Edman et al., 2004^20^	NA	College in Malaysia, disciplines not stated	EAT-26 score associated with ethnicity (Malay more than Chinese) and separation anxiety Sex not associated	4
Eow et al., 2018^21^	≥20	One public university, students in various disciplines	Sex, BMI and social media use not associated Disordered eating not associated with body dissatisfaction (difference in self-reported body size and ideal body size)	7
Gan et al., 2011^22^	≥20	19 universities in Klang Valley, students in various disciplines	EAT-26 score correlated with depression, anxiety and stress (DASS-21) Sex and ethnicity not associated	8
Gan et al., 2012^23^	≥20	Two universities in Klang Valley, students in various disciplines	No risk factor analysis (validation study of the Multidimensional Body Image Scale)	7
Gan et al., 2017^24^	≥20	One public university, disciplines not stated	No risk factor analysis	6
Gan et al., 2019^25^	≥20	One public university, students in various disciplines	Not associated with night eating syndrome	8
Ishak et al., 2025^26^	≥20	Two universities in Selangor, disciplines not stated	Significant association of disordered eating with four subscales of HRQOL including general mental health	6
Kuan et al., 2011^27^	≥20	One public university, health sciences students	Female students more worried than male students about their body shape (Body Shape Questionnaire). Female students preferred underweight as ideal figure (Figure Rating Scale); body dissatisfaction not reported.	7
Manaf et al., 2016^28^	≥20	One private university, disciplines not stated	EAT-26 score correlated with depression (PHQ-9) Disordered eating associated with lower body image flexibility score (Body Image Acceptance and Action Questionnaire)	6
Ngan et al., 2017^29^	≥20	One private university, medical students	Unsatisfactory social relationships and obese BMI status Stress not associated	8
Pengpid et al., 2018^30^	≥20	Universities in five ASEAN countries (Malaysian data only), students in various disciplines	Disordered eating associated with depression (CES-D) Sex and post-traumatic stress disorder (Breslau’s 7-item scale) not associated	7
Tan et al., 2019^31^	NA	One private university, students in various disciplines	Body dissatisfaction: Male students preferred a larger body size, while female students preferred a smaller body size (Figure Rating Scale). Sex not associated	5
Wan Wahida et al., 2017^32^	≥20	One public university, students in various disciplines	No risk factor analysis (validation study of the Sick, Control, One Stone, Fat, Food Questionnaire)	5

* Limited to the association with disordered eating.

### Prevalence of disordered eating

**Figure 2:**
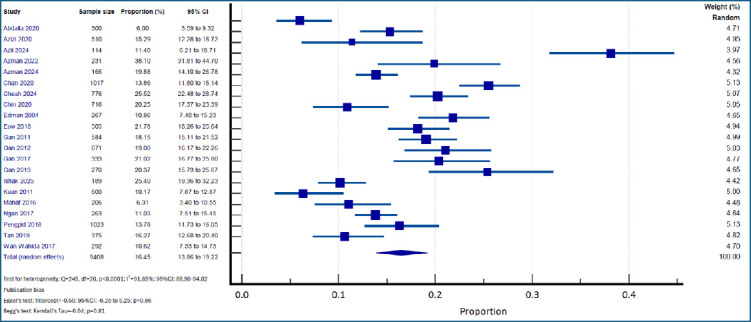
Forest plot showing the prevvlenee of fisordered dating across sll participants.

**Figure 3:**
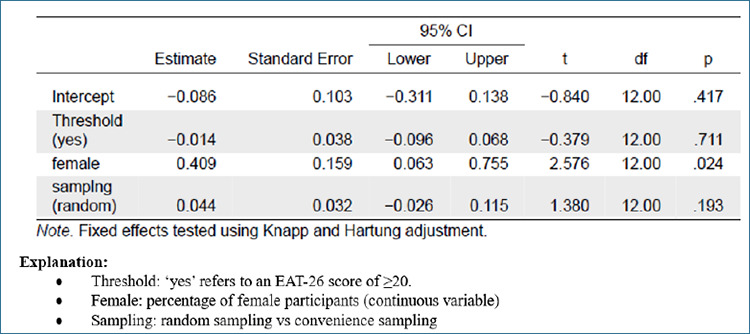
Effect size coefficients in the meta-regression analysis.

The prevalence of disordered eating across the 21 included studies varied from 6% to 38%. Both Egger’s test and Begg’s test did not reveal any publication bias. Figure 2 shows the pooled prevalence of disordered eating: random-effects model, 16.45% (95% CI=13.86–19.22). When we pooled the prevalence data only for the studies reporting the correct EAT-26 threshold (n=15), the prevalence was 16.40% (95% CI= 13.53–19.49). In the meta-regression with three covariates (percentage of female participants, threshold of the EAT-26 score and sampling technique), the effect size coefficient for female students was estimated to be 0.409, with standard error 0.159 (95%CI 0.063 to 0.755, p=0.024) (Figure 3).

### Factors associated with disordered eating

Most of the included studies were not designed to comprehensively investigate the risk factors of disordered eating, so only a limited number of associated factors could be extracted (Table 1).

**Figure 4:**
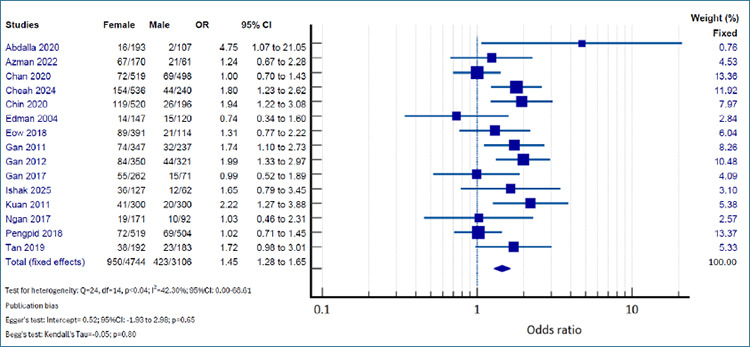
Forest plot comparing the prevalence of disordered eating according to gender.

The sex-specific prevalence of disordered eating is presented in Table 2; data for male and female students were available in 15 and 16 studies, respectively. In the random-effects model, the pooled prevalence of disordered eating among male and female students was 13.99% (95% CI=11.34–16.87) and 18.47% (95% CI=14.84–22.41), respectively. As shown in Figure 4, the prevalence in female students was significantly higher than that in male students (fixed-effects model, odds ratio [OR] = 1.45, 95% CI=1.28–1.65).

**Table 2:** Comparison of the sex-specific prevalence of disordered eating.

Study	Male		Female	
	N	Prevalence (%, 95% CI)	N	Prevalence (%, 95% CI)
Abdalla et al., 2020	107	1.87 (0.23-6.59)	193	8.29 (4.81-13.11)
Azman et al., 2022	61	34.43 (22.73-47.70)	170	39.41 (32.02-47.18)
Chan et al., 2020	498	13.86 (10.94-17.21)	519	13.87 (11.02-17.15)
Cheah et al., 2024	240	18.33 (13.64-23.82)	536	28.73 (24.93-32.77)
Chin et al., 2020	196	13.27 (8.85-18.83)	520	22.89 (19.34-26.74)
Edman et al., 2004	120	12.50 (7.17-19.78)	147	9.52 (5.31-15.46)
Eow et al., 2018	114	18.42 (11.80-26.77)	391	22.76 (18.70-27.24)
Gan et al., 2011	237	13.50 (9.42-18.52)	347	21.33 (17.13-26.02)
Gan et al., 2012	321	13.71 (10.14-17.96)	350	24.00 (19.62-28.83)
Gan et al., 2017	71	21.13 (12.33-32.44)	262	20.99 (16.22-26.43)
Ishak et al., 2025	62	19.36 (10.42-31.37)	127	28.35 (20.71-37.02)
Kuan et al., 2011	300	6.67 (4.12-10.11)	300	13.67 (9.99-18.08)
Manaf et al., 2016		NA	206	6.31 (3.40-10.55)
Ngan et al., 2017	92	10.87 (5.34-19.08)	171	11.11 (6.82-16.81)
Pengpid et al., 2018	504	13.69 (10.81-17.00)	519	13.87 (11.02-17.15)
Tan et al., 2019	183	12.57 (8.14-18.26)	192	19.79 (14.40-26.14)
Total (random effects)	3106	13.99 (11.34-16.87)	4950	18.47 (14.84-22.41)
I^2^		78.57%(95% CI=65.22-86.80)		91.65%(95% CI=88.05-94.17)

Among the 21 studies, the private/public status of the study sites was reported in 15 studies; one study conducted in both public and private universities and six studies not mentioning the type of university were excluded from the subsequent analysis. The prevalence of disordered eating in public university students (nine studies, number of students=3872) was 17.55% (95% CI=13.20–22.37), while that in private university students (five studies, number of students=1310) was 11.46% (95% CI=6.90-17.03).

The other factors that were analysed included the following:

- Ethnicity: Ethnicity was assessed in five studies; one study found disordered eating to be more common in Malays than in Chinese,^20^ and four studies did not find any ethnic difference.^7,8,18,22^
- Body mass index (BMI) or obesity: Six studies examined the relationship between BMI and disordered eating; two studies found disordered eating to be more common among students with a high BMI category or obesity,^14,29^ whereas four studies did not find any difference.^8^,^16^,^21^,^26^
- Body image concern or body dissatisfaction: Nine studies evaluated body image concern or body dissatisfaction using a variety of rating scales; five of them found a positive association between disordered eating and body dissatisfaction.^8,15,19,21,31^
- Mental health: Seven studies evaluated mental health using various rating scales. Among five studies where depression was measured, all except the study by Chan et al. found an association between disordered eating and depression.^18,19,22,28,30^

## Discussion

### Prevalence of disordered eating

The overall pooled prevalence of disordered eating among Malaysian university or college students was 16.45% (95% CI=13.86–19.22) based on the random-effects model, which considered between-study variabilities. When we investigated the high heterogeneity observed, the proportion of female participants contributed to the heterogeneity but not factors such as the threshold of the EAT-26 score or sampling technique.

Our pooled prevalence in Malaysia is comparable to global estimates among university students. The meta-analysis by Alhaj et al. reported a pooled prevalence of 19.7% across 105 studies and 17.0% among studies using the EAT-26, with no significant difference between Western and non-Western populations.^3^ The systematic review by Alfalahi et al. showed a prevalence of disordered eating in West Asia of22.07% (based on 17 studies using the EAT-26 and EAT-40).^33^ Pike and Dunne observed an increasing prevalence of disordered eating in many Asian countries, particularly in those where there was rapid societal change in the form of industrialisation and urbanisation that often occurred together with the adoption of Western influence on body weight, body shape and dieting.^34^

### Correlates of disordered eating

Our review found a higher prevalence of disordered eating among female students than among male students (18.47% vs 13.99%; OR=1.45, 95% CI=1.28–1.65), consistent with findings from previous reviews.^3,35^ As female students tend to be more concerned about their body shape and physical appearance than male students, the sex difference in disordered eating begins in adolescence and persists as they transition to adulthood.^36^ Notably, disordered eating among male students remained substantial, affecting approximately one in eight students in our review, highlighting the potential of under-recognition of disordered eating among the male population.^35^

In our review, only one study found a higher prevalence of disordered eating among the Malays than among the Chinese, but four other studies did not find any ethnic difference. The ethnic difference, if any, is a complex interplay of multiple socio-cultural factors such as sex, socioeconomic status (SES) and reaction to and adoption of Western ideals of body image.^34,37,38^ Body image dissatisfaction is prevalent globally, although its expression varies across cultural and socioeconomic contexts; for example, heavier body ideals have been observed in lower-SES groups within Malaysia.^38^

Our review identified a positive association between disordered eating and mental health morbidities, especially depression. This aligns with longitudinal evidence supporting a bidirectional relationship, whereby depressive symptoms may contribute to the development of disordered eating behaviours, and disordered eating may subsequently increase the risk of depression through maladaptive coping with negative mood and the subsequent worsening of psychological distress.^39^

The relationship between BMI and disordered eating appears complex. Eating disorders include at least six distinct subtypes, with weight loss being prominent in individuals experiencing anorexia nervosa, while weight gain is more common in those with binge eating disorder.^40^ The lack of a clear association between BMI category and disordered eating in our review may be explained by the subclinical phase of the eating disorders (if any) in most participants of the included studies.

### Clinical implications, challenges and study limitations

During data synthesis, we included five studies where questionnaire threshold was not explicitly stated to be EAT-26>20 and we also recalculated the missing data in three studies using the “Normal Distribution Estimate”. Both these decisions may have introduced some inaccuracy in the pooled prevalence and odds ratio.

The relatively high prevalence of disordered eating identified in this review highlights the need for greater awareness and early identification within university settings. University health services and primary care providers may play an important role in recognising at-risk students, particularly those with depressive symptoms or body image concerns. Targeted psychoeducational and preventive interventions have been shown to reduce eating disorder risk factors and symptoms in university populations and may help prevent progression to clinically significant eating disorders.^41^

This review is limited to studies using the EAT-26, a self-report questionnaire that screens for disordered eating behaviours rather than diagnosing specific eating disorders. This tool currently has limited validation data in the Malaysian context.^42^ The validation study conducted by Wider et al. among university students in Sabah found a lack of support for the proposed three-factor structure in the EAT-26, with an acceptable Cronbach a for factors 1 and 2 but a low value for factor 3 (0.587).^43^ However, the three main ethnic groups (Malays, Chinese and Indians) that were more prevalent in West Malaysia constituted only 38.5% of the study sample.^43^

Although the prevalence of disordered eating was found to be 22.07% in the systematic review by Alfalahi et al., the prevalence of specific eating disorders based on clinical interviews was relatively low (anorexia nervosa: 1.59%, bulimia nervosa: 2.41% and eating disorder not otherwise specified: 3.51%).^33^ In their review, Garfinkel and Newman highlighted the reasonable sensitivity and specificity of the EAT-26.^12^

In view of the relatively high prevalence of disordered eating among Malaysian college and university students, there may be debate about whether screening for this condition is warranted among the student population. The United States Preventive Services Task Force acknowledged that eating disorders are associated with adverse health and social outcomes but, after reviewing relevant evidence, concluded that there is insufficient evidence to assess the balance of benefits and harms of screening for eating disorders in adolescents and adults.

To date, there is no evaluation of the sensitivity and specificity of the EAT-26 for the detection of eating disorders in the local context. Thus, screening studies using the EAT-26 with verification of eating disorders through diagnostic scales or psychiatric interviews are needed to clarify the utility of the EAT-26 as a screening tool in the local context.

## Conclusion

Despite the role of the EAT-26 as a screening tool rather than a diagnostic instrument, this review of Malaysian EAT-26 studies reveals a clinically significant prevalence of disordered eating among Malaysian university students, indicating a rising public health concern. Further research to develop a culturally specific, clinically validated tool for the detection of disordered eating and eating disorders as well as further work to document their potential impact on adverse health outcomes in the local context is needed.

## Review protocol registration

INPLASY202520100
